# Editorial: Cellular Mechanisms of Ototoxicity

**DOI:** 10.3389/fncel.2018.00075

**Published:** 2018-03-27

**Authors:** Peter S. Steyger, Lisa L. Cunningham, Carlos R. Esquivel, Kelly L. Watts, Jian Zuo

**Affiliations:** ^1^Oregon Hearing Research Center, Oregon Health & Science University, Portland, OR, United States; ^2^National Center for Rehabilitative Auditory Research, VA Portland Health Care System, Portland, OR, United States; ^3^National Institute on Deafness and Other Communication Disorders, Bethesda, MD, United States; ^4^Hearing Center of Excellence, J-9, Defense Health Agency, Joint Base San Antonio, San Antonio, TX, United States; ^5^Naval Submarine Medical Research Laboratory, Naval Submarine Base New London, Groton, CT, United States; ^6^zCore Business Solutions, Round Rock, TX, United States; ^7^Department of Developmental Neurobiology, St. Jude Children's Research Hospital, Memphis, TN, United States

**Keywords:** ototoxicity, cochleotoxicity, vestibulotoxicity, cytotoxicity, otoprotection

Spoken language allows most people^1^ to communicate with loved ones, friends and/or colleagues. Those who lose their ability to hear may experience isolation, depression, loss of mental acuity, and diminishing integration within wider society (Lin et al., 2013). Children unable to acquire the full range of listening and spoken language skills that are present in peers with normal hearing can face delayed academic, linguistic and psychosocial milestones (Gurney et al., 2007; Dedhia et al., 2013). These outcomes in turn can reduce professional opportunities, emotional connectivity, and intimacy with others (Jarvelin et al., 1997; Hornsby and Kipp, 2016). On an evolutionary level, hearing loss reduces the perception of environmental auditory cues associated with beneficial or detrimental outcomes (e.g., localizing sounds associated with mating or predators). Thus, the ability to hear well is crucial. Loss of vestibular function is similarly debilitating, with reduced mobility and integration within society, often leading to depression, and cognitive decline (Smith and Darlington, 2013; Smith and Zheng, 2013). Both peripheral auditory and vestibular sensory organs are closely located anatomically, within the inner ear, and both rely on sensory hair cells to detect sound, gravity, rotation, and acceleration.

Our ability to hear and maintain postural control can be affected by many factors, including congenital genetic mutations, aging, noise exposure, trauma, selected infections, and a variety of environmental exposures and pharmaceutical interventions. Ototoxicity refers to damage to the inner ear, specifically cochlear and vestibular structures and functions, due to exposure to pharmaceuticals, chemicals, and/or ionizing radiation. Ototoxic compounds can also damage the auditory and/or vestibular neural pathways to the brainstem, and beyond to the auditory cortex. However, we generally define ototoxicity as affecting the peripheral inner ear, inducing auditory dysfunction (cochleotoxicity) or vestibular deficits (vestibulotoxicity).

This Research Topic, *Cellular Mechanisms in Ototoxicity*, contains both original research articles and focused reviews on current and fundamental questions of how ototoxic substances damage the inner ear, and it includes therapeutic approaches to prevent or repair ototoxic injury. Aminoglycoside antibiotics (Jiang et al.) and platinum-based drugs (Sheth et al.) are the primary ototoxins reviewed here. Intriguingly, a more recent study demonstrated that cisplatin (Breglio et al., 2017), like aminoglycosides (Aran et al., 1999), is retained by cochlear tissues for extended periods of time, likely contributing to the intracellular mechanisms of cytotoxicity and cell death reviewed here (Francis and Cunningham; Nicholas et al.) that can continue after cessation of drug administration. Several articles discussed how candidate otoprotectants revealed and/or ameliorated postulated mechanisms of ototoxicity (Fransson et al.; Jadali et al.; Kirkwood et al.; Wiedenhoft et al.).

These studies will inform future research aimed at: (i) characterizing the mechanisms underlying the damage caused by individual ototoxins, including a recently-identified ototoxin (Crumling et al.), elucidating the causes of drug-induced cochleotoxicity and vestibulotoxicity (Sultemeier and Hoffman), and (ii) identifying novel pharmaceutical interventions to reduce ototoxicity (Noack et al.; O'Sullivan et al.; Kim et al.). One novel otoprotective strategy described here is the delivery of steroids using magnetic nanoparticles to ameliorate cisplatin-induced hearing loss (Ramaswamy et al.).

In addition, candidate strategies that are otoprotective in one species can inadvertently potentiate ototoxicity in another, or when translated from an *in vitro* model to an *in vivo* model (Majumder et al.; Yang et al.). Determining the structure-activity relationships (i.e., how chemical structures affect the efficacy and/or safety) of candidate otoprotectants and their derivatives against ototoxicity and noise-induced hearing loss will accelerate the translation of candidate otoprotectants into clinical trials, similar to those described since these articles were published (Chowdhury et al., 2017; Kenyon et al., 2017). This arena is rapidly advancing with several promising lines of drug discovery and optimization strategies.

Inflammation can potentiate ototoxicity, and four articles discussed the role of inflammation during ototoxic injury and subsequent recovery (Jiang et al.; Kalinec et al.; Mwangi et al.; Wood and Zuo). Another area of increasing interest is the role of microRNAs in disease and infections, including ototoxicity and noise-induced hearing loss (Prasad and Bondy). Additional areas where we can expect new research include: (i) antibiotic stewardship and (ii) inadvertent protection of bacteria and tumors by otoprotectants. National antibiotic stewardship programs aim to reduce the clinical (and agricultural) use of antibiotics to slow the evolution of antibiotic-resistant microbes. Stewardship programs will also alter current clinical prescribing practices to sustain the bactericidal efficacy of existing antibiotics. Ultimately, multidrug-resistant bacteria frequently remain susceptible only to ototoxic aminoglycoside antibiotics, and dosing with the ototoxic aminoglycosides could increase sharply in the future. Secondly, candidate otoprotectants need to be screened to ensure they do not inadvertently protect bacteria or tumor cells from the crucial cytotoxic effects of aminoglycosides and platinum-based drugs.

Currently, sensorineural hearing loss is irreversible, and there is a great need to protect the hearing of patients, workers and others exposed to cochleotoxic and vestibulotoxic substances. Developing efficacious otoprotective strategies is an area of intense research, and progress will accelerate as the mechanisms underlying ototoxicity are identified. Otoprotection can presumably be more readily achieved to protect those with normal hearing, while other research groups continue longer-term strategies to develop a viable, and much-needed, intervention to restore hearing for those with existing hearing loss. Protection, rehabilitation, repair and ultimately restoration of the ability to hear and coordinate body posture will enhance the quality of life for millions of individuals and reap enormous socioeconomic benefits.

On behalf of all the authors in this Research Topic, we want to thank the Hearing Center of Excellence (headquartered in San Antonio, Texas) for ensuring these contributions to the scientific literature are freely accessible to all, enabling scientists to best direct our research efforts as we move toward establishing a future free of ototoxicity.

## Author contributions

PS, LC, and JZ wrote the original draft, and all authors revised and approved this editorial.

### Conflict of interest statement

The authors declare that the research was conducted in the absence of any commercial or financial relationships that could be construed as a potential conflict of interest.

## Footnotes

^1^ Manual languages, which are rich in expression of thoughts, and feelings, are utilized by ~1% of the population, often due severe to profound hearing loss. This editorial focused on how acquired hearing loss can affect spoken communication.

## References

[B1] Aran J. M. Erre J. P. Lima Da Costa D. Debbarh I. Dulon D. (1999). Acute and chronic effects of aminoglycosides on cochlear hair cells. Ann. N. Y. Acad. Sci. 884, 60–68. 10.1111/j.1749-6632.1999.tb08636.x 10842584

[B2] Breglio A. M. Rusheen A. E. Shide E. D. Fernandez K. A. Spielbauer K. K. Mclachlin K. M.. (2017). Cisplatin is retained in the cochlea indefinitely following chemotherapy. Nat. Commun. 8, 1654. 10.1038/s41467-017-01837-1 29162831 PMC5698400

[B3] Chowdhury S. Owens K. N. Herr R. J. Jiang Q. Chen X. Johnson G.. (2017). Phenotypic Optimization of Urea-Thiophene Carboxamides To Yield Potent, Well Tolerated, and Orally Active Protective Agents against Aminoglycoside-Induced Hearing Loss. J. Med. Chem. 61, 84–97. 10.1021/acs.jmedchem.7b00932 28992413 PMC5889090

[B4] Dedhia K. Kitsko D. Sabo D. Chi D. H. (2013). Children with sensorineural hearing loss after passing the newborn hearing screen. JAMA Otolaryngol. Head Neck Surg. 139, 119–123. 10.1001/jamaoto.2013.1229 23328914

[B5] Gurney J. G. Tersak J. M. Ness K. K. Landier W. Matthay K. K. Schmidt M. L.. (2007). Hearing loss, quality of life, and academic problems in long-term neuroblastoma survivors: a report from the Children's Oncology Group. Pediatrics 120, e1229–e1236. 10.1542/peds.2007-0178 17974716

[B6] Hornsby B. W. Kipp A. M. (2016). Subjective ratings of fatigue and vigor in adults with hearing loss are driven by perceived hearing difficulties not degree of hearing loss. Ear Hear. 37, e1–e10. 10.1097/AUD.0000000000000203 26295606 PMC6681455

[B7] Järvelin M. R. Mäki-Torkko E. Sorri M. J. Rantakallio P. T. (1997). Effect of hearing impairment on educational outcomes and employment up to the age of 25 years in northern Finland. Br. J. Audiol. 31, 165–175. 10.3109/03005364000000019 9276099

[B8] Kenyon E. J. Kirkwood N. K. Kitcher S. R. O'Reilly M. Derudas M. Cantillon D. M.. (2017). Identification of ion-channel modulators that protect against aminoglycoside-induced hair cell death. JCI Insight 2:96773. 10.1172/jci.insight.96773 29263311 PMC5752279

[B9] Lin F. R. Yaffe K. Xia J. Xue Q. L. Harris T. B. Purchase-Helzner E.. (2013). Hearing loss and cognitive decline in older adults. JAMA Intern. Med. 173, 293–299. 10.1001/jamainternmed.2013.1868 23337978 PMC3869227

[B10] Smith P. F. Darlington C. L. (2013). Personality changes in patients with vestibular dysfunction. Front. Hum. Neurosci. 7:678. 10.3389/fnhum.2013.00678 24194706 PMC3810789

[B11] Smith P. F. Zheng Y. (2013). From ear to uncertainty: vestibular contributions to cognitive function. Front. Integr. Neurosci. 7:84. 10.3389/fnint.2013.00084 24324413 PMC3840327

